# Supplementary material for: Meal Patterns and Changes in Cardiometabolic Risk Factors in Children: A Longitudinal Analysis
Source: Nutrients. 2020 Mar 18;12(3):799. doi: 10.3390/nu12030799 (PMC7146132; doi:10.3390/nu12030799)
Supplement: Supplementary file 1 [file nutrients-12-00799-s001.pdf]

Figure S1. Flowchart for population section

Table S1. P values for interaction between sex and energy/macronutrients intake at different meals for changes in cardiometabolic risk score

Table S2. P values for interaction between intervention and energy/macronutrients intake at different meals for changes in cardiometabolic risk score

Table S3. Differences in baseline characteristics between children who were included in the analysis and those who had missing or abnormal diet data

Table S4. Characteristics by meal patterns

Table S5. Meal patterns and changes in cardiometabolic risk factors in children

Table S6. Change in macronutrients intake at different meals and change in cardiometabolic risk score

Table S7. Meal patterns and changes in cardiometabolic risk factors in children in the control group

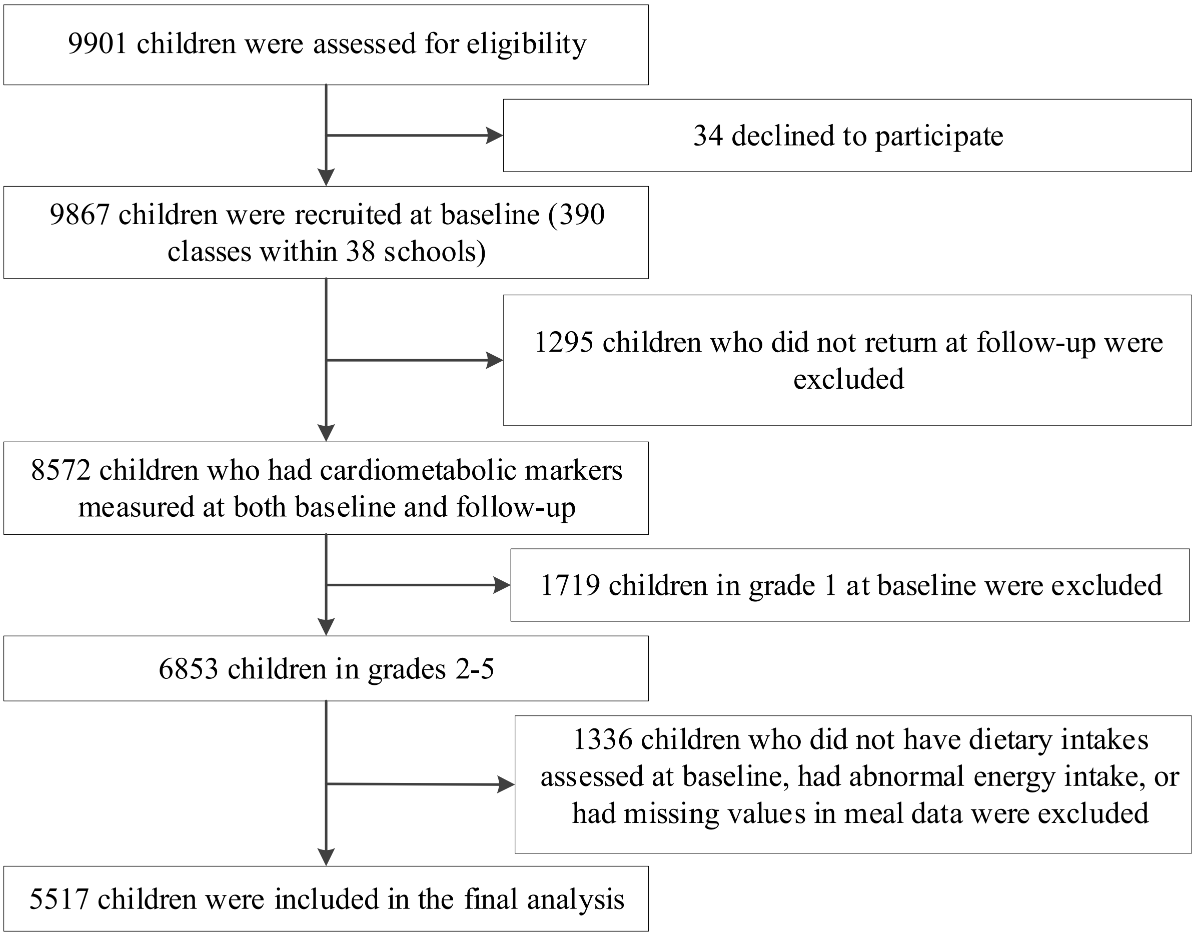
9901 children were assessed for eligibility
34 declined to participate
9867 children were recruited at baseline (390 classes within 38 schools)
1295 children who did not return at follow-up were excluded
8572 children who had cardiometabolic markers measured at both baseline and follow-up
1719 children in grade 1 at baseline were excluded
6853 children in grades 2-5
1336 children who did not have dietary intakes assessed at baseline, had abnormal energy intake, or had missing values in meal data were excluded
5517 children were included in the final analysis

Figure S1. Flowchart for population section

Table S1. P values for interaction between sex and energy/macronutrients intake at different meals for changes in cardiometabolic risk score*

	P interaction for sex and dietary factor-Model 1	P interaction for sex and dietary factor-Model 2	P interaction for sex and dietary factor-Model 3
Meal pattern	0.73	0.73	0.74
Energy from breakfast	0.46	0.81	0.80
Energy from lunch	0.49	0.75	0.79
Energy from dinner	0.52	0.43	0.39
Energy from snacks	0.91	0.89	0.86
Carbohydrate at breakfast	0.0042	0.0052	0.0080
Carbohydrate at lunch	0.0079	0.0254	0.0195
Carbohydrate at dinner	0.16	0.22	0.21
Carbohydrate at snacks	0.21	0.21	0.21
Protein at breakfast	0.17	0.10	0.10
Protein at lunch	0.0513	0.0145	0.0150
Protein at dinner	0.74	0.59	0.62
Protein at snacks	0.61	0.59	0.64
Fat at breakfast	0.14	0.17	0.22
Fat at lunch	0.48	0.85	0.86

Fat at dinner	0.0030	0.0091	0.0100
Fat at snacks	0.0771	0.0109	0.0102

*GLM was used to test the interaction between sex and energy/macronutrient intake at different meals for changes in the cardiometabolic risk score. Model 1 was adjusted for classes in schools as clustering effects and characteristics of individuals including age, sex, and CMRS at baseline as fixed effects. Model 2 was adjusted for Model 1 plus puberty, grade, intervention, BMI, physical activity, and energy intake at baseline as fixed effects. Model 3 was adjusted for Model 2 plus birthweight, household income, mother's education, father's education, mother's BMI, and father's BMI as fixed effects. We used the Benjamin-Hochberg procedure was used to control the false discovery rate at level 5% for multiple comparisons with the P-value cut-off point of significance was 0.0029 for model 3.

Table S2. P values for interaction between intervention and energy/macronutrients intake at different meals for changes in cardiometabolic risk score*

	P interaction for intervention and dietary factor-Model 1	P interaction for intervention and dietary factor-Model 2	P interaction for intervention and dietary factor-Model 3
Meal pattern	0.0389	0.0201	0.0176
Energy from breakfast	0.74	0.89	0.87
Energy from lunch	0.0067	0.0092	0.0060
Energy from dinner	0.0531	0.0460	0.0578
Energy from snacks	0.30	0.70	0.75
Carbohydrate at breakfast	0.70	0.68	0.70
Carbohydrate at lunch	0.04	0.14	0.16
Carbohydrate at dinner	0.43	0.24	0.25
Carbohydrate at snacks	0.25	0.25	0.25
Protein at breakfast	0.95	0.94	0.97
Protein at lunch	0.07	0.10	0.12
Protein at dinner	0.90	0.79	0.75
Protein at snacks	0.83	0.98	0.97
Fat at breakfast	0.13	0.05	0.07
Fat at lunch	0.05	0.19	0.19
Fat at dinner	0.43	0.33	0.39
Fat at snacks	0.14	0.17	0.19

*GLM was used to test the interaction between intervention and energy/macronutrient intake at different meals for changes in the cardiometabolic risk score. Model 1 was adjusted for

classes in schools as clustering effects and characteristics of individuals including age, sex, and CMRS at baseline as fixed effects. Model 2 was adjusted for Model 1 plus puberty, grade, intervention, BMI, physical activity, and energy intake at baseline as fixed effects. Model 3 was adjusted for Model 2 plus birthweight, household income, mother's education, father's education, mother's BMI, and father's BMI as fixed effects. We used the Benjamin-Hochberg procedure was used to control the false discovery rate at level 5% for multiple comparisons with the P-value cut-off point of significance was 0.0029 for model 3.

Table S3. Differences in baseline characteristics between children who were included in the analysis and those who had missing or abnormal diet data

	All	Included	Lost	P-value*
Age (years)	9.55 ± 1.18[†]	9.55 ± 1.18	9.57 ± 1.17	0.45
BMI (kg/m^2)	17.24 ± 3.19	17.20 ± 3.18	17.43 ± 3.21	0.0146
WC (cm)	58.66 ± 8.92	58.48 ± 8.84	59.40 ± 9.20	0.0007
PBF (%)	23.95 ± 4.88	23.93 ± 4.84	24.04 ± 5.06	0.48
SBP (mm Hg)	100.49 ± 10.80	100.67 ± 10.86	99.76 ± 10.50	0.0056
DBP (mm Hg)	64.03 ± 9.09	64.18 ± 9.08	63.43 ± 9.07	0.0059
MAP (mm Hg)	76.17 ± 8.89	76.33 ± 8.92	75.51 ± 8.74	0.0022
TC (mmol/L)	4.09 ± 0.78	4.08 ± 0.79	4.12 ± 0.76	0.14
HDL-C (mmol/L)	1.48 ± 0.31	1.47 ± 0.30	1.50 ± 0.32	0.0003
LDL-C (mmol/L)	2.13 ± 0.63	2.12 ± 0.63	2.15 ± 0.61	0.12
TG (mmol/L)	0.82 ± 0.45	0.82 ± 0.45	0.81 ± 0.43	0.54
Fasting glucose (mmol/L)	4.53 ± 0.56	4.52 ± 0.56	4.59 ± 0.57	0.0001
Log insulin	1.66 ± 0.61	1.65 ± 0.61	1.70 ± 0.62	0.0198
Log HOMA-IR	-2.83 ± 0.65	-2.85 ± 0.65	-2.79 ± 0.65	0.0039
CMRS	-0.23 ± 2.41	-0.22 ± 2.40	-0.23 ± 2.44	0.94
Energy (kcal/day)	1269.80 ± 523.62	1271.75 ± 585.29	1262.01 ± 34.32	0.54
Physical activity (MET)	636.41 ± 560.22	628.60 ± 561.34	667.57 ± 554.81	0.0214
Sex				<0.0001

Boys	3475 (50.8)‡	2703 (49.5)	772 (56.4)	
Girls	3359 (49.2)	2762 (50.5)	597 (43.6)	
Puberty				0.06
No	6313 (92.4)	5032 (92.1)	1281 (93.6)	
Yes	521 (7.6)	433 (7.9)	88 (6.4)	
Grade				0.16
Two	1888 (27.6)	1535 (28.1)	353 (25.8)	
Three	1881 (27.5)	1498 (27.4)	383 (28.0)	
Four	1900 (27.8)	1504 (27.5)	396 (28.9)	
Five	1165 (17.0)	928 (17.0)	237 (17.3)	
Birth weight				0.0486
<2500 g	228 (3.3)	188 (3.4)	40 (2.9)	
2500-3999 g	5107 (74.7)	4353 (79.7)	754 (55.1)	
≥4000 g	573 (8.4)	502 (9.2)	71 (5.2)	
Missing	926 (13.5)	422 (7.7)	504 (36.8)	
Mother's BMI				0.60
<24 kg/m^2	4909 (71.8)	4181 (76.5)	728 (53.2)	
24-27.9 kg/m^2	1049 (15.3)	902 (16.5)	147 (10.7)	
≥28 kg/m^2	149 (2.2)	126 (2.3)	23 (1.7)	
Missing	727 (10.6)	256 (4.7)	471 (34.4)	
Father's BMI				0.57
<24 kg/m^2	3387 (49.6)	2894 (53.0)	493 (36.0)	
24-27.9 kg/m^2	2173 (31.8)	1856 (34.0)	317 (23.2)	
≥28 kg/m^2	547 (8.0)	459 (8.4)	88 (6.4)	
Missing	727 (10.6)	256 (4.7)	471 (34.4)	

Mother's education				0.0001
<7 years	717 (10.5)	640 (11.7)	77 (5.6)	
7-12 years	3883 (56.8)	3320 (60.8)	563 (41.1)	
≥13 years	1373 (20.1)	1137 (20.8)	236 (17.2)	
Missing	861 (12.6)	368 (6.7)	493 (36.0)	
Father's education				0.0020
<7 years	416 (6.1)	365 (6.7)	51 (3.7)	
7-12 years	4011 (58.7)	3449 (63.1)	562 (41.1)	
≥13 years	1567 (22.9)	1302 (23.8)	265 (19.4)	
Missing	840 (12.3)	349 (6.4)	491 (35.9)	
Household income per month				0.0016
<750 RMB	702 (10.3)	610 (11.2)	92 (6.7)	
751-1500 RMB	1962 (28.7)	1698 (31.1)	264 (19.3)	
1501-2500 RMB	1609 (23.5)	1384 (25.3)	225 (16.4)	
≥2501 RMB	1631 (23.9)	1352 (24.7)	279 (20.4)	
Missing	930 (13.6)	421 (7.7)	509 (37.2)	
Intervention				0.0078
No	3074 (45.0)	2502 (45.8)	572 (41.8)	
Yes	3760 (55.0)	2963 (54.2)	797 (58.2)	

*T-test was used to test the difference of continuous variables between boys and girls and Chi-square for categorical variables.

†All such data were mean ± standard deviation.

‡All such data were frequency (percentage).

Table S4. Characteristics by meal patterns

	Meal pattern					P-value*
	Balanced	Breakfast dominant	Lunch dominant	Dinner dominant	Snack dominant	
Birth weight						0.71
<2500 g	41 (3.0)[†]	56 (3.6)	51 (4.0)	36 (3.8)	8 (2.4)	
2500-3999 g	1131 (81.9)	1232 (78.3)	973 (76.9)	783 (81.6)	277 (82.2)	
≥4000 g	106 (7.7)	157 (10.0)	140 (11.1)	73 (7.6)	29 (8.6)	
Missing	103 (7.5)	129 (8.2)	102 (8.1)	67 (7.0)	23 (6.8)	
Mother's BMI						0.41
<24 kg/m^2	1070 (77.5)	1165 (74.0)	971 (76.7)	746 (77.8)	266 (78.9)	
24-27.9 kg/m^2	232 (16.8)	278 (17.7)	217 (17.1)	141 (14.7)	44 (13.1)	
≥28 kg/m^2	23 (1.7)	44 (2.8)	24 (1.9)	31 (3.2)	8 (2.4)	
Missing	56 (4.1)	87 (5.5)	54 (4.3)	41 (4.3)	19 (5.6)	
Father's BMI						0.30
<24 kg/m^2	777 (56.3)	799 (50.8)	653 (51.6)	506 (52.8)	182 (54.0)	

24-27.9 kg/m^2	446 (32.3)	533 (33.9)	452 (35.7)	340 (35.5)	108 (32.0)	
≥28 kg/m^2	102 (7.4)	155 (9.8)	107 (8.5)	72 (7.5)	28 (8.3)	
Missing	56 (4.1)	87 (5.5)	54 (4.3)	41 (4.3)	19 (5.6)	
Mother's education						0.55
<7 years	136 (9.8)	248 (15.8)	118 (9.3)	90 (9.4)	52 (15.4)	
7-12 years	855 (61.9)	930 (59.1)	767 (60.6)	580 (60.5)	207 (61.4)	
≥13 years	306 (22.2)	279 (17.7)	301 (23.8)	226 (23.6)	52 (15.4)	
Missing	84 (6.1)	117 (7.4)	80 (6.3)	63 (6.6)	26 (7.7)	
Father's education						0.87
<7 years	81 (5.9)	129 (8.2)	72 (5.7)	52 (5.4)	33 (9.8)	
7-12 years	863 (62.5)	1012 (64.3)	789 (62.3)	585 (61.0)	219 (65.0)	
≥13 years	358 (25.9)	321 (20.4)	331 (26.1)	260 (27.1)	60 (17.8)	
Missing	79 (5.7)	112 (7.1)	74 (5.8)	62 (6.5)	25 (7.4)	
Household income per month						0.73
<750 RMB	155 (11.2)	190 (12.1)	143 (11.3)	99 (10.3)	28 (8.3)	
751-1500 RMB	418 (30.3)	468 (29.7)	406 (32.1)	301 (31.4)	117 (34.7)	

1501-2500 RMB	339 (24.5)	402 (25.5)	321 (25.4)	253 (26.4)	81 (24.0)	
≥2501 RMB	368 (26.6)	382 (24.3)	304 (24.0)	239 (24.9)	80 (23.7)	
Missing	101 (7.3)	132 (8.4)	92 (7.3)	67 (7.0)	31 (9.2)	
Intervention						0.81
No	645 (46.7)	686 (43.6)	600 (47.4)	426 (44.4)	163 (48.4)	
Yes	736 (53.3)	888 (56.4)	666 (52.6)	533 (55.6)	174 (51.6)	

*The Chi-square test was used to test the difference of variables across meal patterns.

[†]All such data were frequency (percentage).

Table S5. Meal patterns and changes in cardiometabolic risk factors in children

	Meal pattern					P-value*
	Balanced	Breakfast dominant	Lunch dominant	Dinner dominant	Snack dominant	
Change in BMI						
Participants	1367	1553	1249	945	334	
β (95% CI), Model 1[†]		0.034 (-0.009, 0.076)	0.036 (-0.009, 0.081)	0.008 (-0.040, 0.057)	-0.011 (-0.081, 0.059)	0.32
β (95% CI), Model 2[‡]		0.044 (0.001, 0.087)	0.044 (-0.001, 0.089)	0.011 (-0.037, 0.059)	-0.007 (-0.076, 0.063)	0.15
Change in WC						
Participants	1360	1549	1245	945	334	
β (95% CI), Model 1		-0.025 (-0.059, 0.009)	0.033 (-0.003, 0.069)	0.004 (-0.034, 0.043)	-0.002 (-0.059, 0.054)	0.0300
β (95% CI), Model 2		-0.011 (-0.045, 0.022)	0.040 (0.005, 0.075)	0.005 (-0.032, 0.043)	-0.015 (-0.070, 0.040)	0.0391
Change in PBF						
Participants	1337	1510	1209	915	326	
β (95% CI), Model 1		0.082 (0.029, 0.134)	0.020 (-0.036, 0.075)	-0.018 (-0.078, 0.042)	-0.027 (-0.114, 0.060)	0.0029
β (95% CI), Model 2		0.075 (0.022, 0.127)	0.008 (-0.047, 0.063)	-0.035 (-0.093, 0.024)	-0.034 (-0.120, 0.052)	0.0019
Change in SBP						
Participants	1361	1551	1246	942	333	
β (95% CI), Model 1		0.106 (0.037, 0.176)	0.087 (0.014, 0.160)	0.070 (-0.009, 0.149)	-0.022 (-0.136, 0.093)	0.0137
β (95% CI), Model 2		0.089 (0.020, 0.157)	0.055 (-0.017, 0.126)	0.050 (-0.026, 0.127)	-0.016 (-0.127, 0.096)	0.0921

Change in DBP						
Participants	1363	1552	1248	943	334	
β (95% CI), Model 1		0.082 (0.012, 0.152)	0.037 (-0.037, 0.110)	-0.010 (-0.089, 0.069)	-0.007 (-0.122, 0.109)	0.09
β (95% CI), Model 2		0.057 (-0.013, 0.127)	0.006 (-0.067, 0.079)	-0.028 (-0.106, 0.051)	-0.002 (-0.116, 0.112)	0.26
Change in MAP						
Participants	1361	1550	1246	943	334	
β (95% CI), Model 1		0.010 (0.030, 0.170)	0.064 (-0.010, 0.137)	0.024 (-0.055, 0.103)	-0.002 (-0.118, 0.113)	0.0451
β (95% CI), Model 2		0.075 (0.006, 0.145)	0.029 (-0.044, 0.101)	0.004 (-0.074, 0.081)	0.003 (-0.110, 0.116)	0.22
Change in TC						
Participants	1283	1460	1175	892	316	
β (95% CI), Model 1		-0.112 (-0.165, -0.059)	-0.023 (-0.079, 0.032)	-0.013 (-0.073, 0.047)	-0.028 (-0.115, 0.060)	0.0003
β (95% CI), Model 2		-0.083 (-0.136, -0.029)	-0.007 (-0.063, 0.048)	-0.011 (-0.070, 0.049)	-0.034 (-0.121, 0.053)	0.0176
Change in HDL-C						
Participants	1284	1459	1175	891	314	
β (95% CI), Model 1		0.042 (-0.034, 0.118)	-0.082 (-0.162, -0.002)	-0.015 (-0.101, 0.070)	0.228 (0.103, 0.354)	<0.0001
β (95% CI), Model 2		0.020 (-0.056, 0.095)	-0.077 (-0.156, 0.001)	-0.005 (-0.089, 0.079)	0.233 (0.110, 0.356)	0.0001
Change in LDL-C						
Participants	1284	1461	1176	891	316	
β (95% CI), Model 1		-0.148 (-0.207, -0.089)	-0.086 (-0.147, -0.024)	-0.012 (-0.079, 0.054)	-0.115 (-0.212, -0.018)	<0.0001

β (95% CI), Model 2		-0.122 (-0.182, -0.063)	-0.073 (-0.135, -0.012)	-0.012 (-0.078, 0.055)	-0.121 (-0.217, -0.024)	0.0002
Change in TG						
Participants	1282	1461	1176	894	317	
β (95% CI), Model 1		-0.058 (-0.127, 0.011)	0.011 (-0.061, 0.083)	0.018 (-0.060, 0.090)	-0.219 (-0.332, -0.106)	0.0004
β (95% CI), Model 2		-0.038 (-0.106, 0.030)	0.003 (-0.067, 0.073)	0.011 (-0.065, 0.086)	-0.214 (-0.324, -0.105)	0.0013
Change in fasting glucose						
Participants	1284	1460	1176	892	317	
β (95% CI), Model 1		0.051 (-0.007, 0.108)	-0.050 (-0.110, 0.010)	0.002 (-0.063, 0.066)	0.070 (-0.024, 0.164)	0.0099
β (95% CI), Model 2		0.028 (-0.030, 0.086)	-0.058 (-0.118, 0.002)	0.004 (-0.060, 0.068)	0.076 (-0.017, 0.170)	0.0189
Change in insulin						
Participants	1132	1278	1035	795	273	
β (95% CI), Model 1		0.092 (-0.021, 0.205)	0.008 (-0.110, 0.126)	0.111 (-0.016, 0.238)	0.060 (-0.126, 0.246)	0.29
β (95% CI), Model 2		0.043 (-0.068, 0.153)	-0.058 (-0.173, 0.056)	0.108 (-0.015, 0.230)	0.050 (-0.130, 0.229)	0.11
Change in HOMA-IR						
Participants	1132	1277	1034	795	273	
β (95% CI), Model 1		0.106 (-0.004, 0.215)	-0.006 (-0.121, 0.109)	0.106 (-0.017, 0.229)	0.081 (-0.100, 0.262)	0.14
β (95% CI), Model 2		0.053 (-0.055, 0.160)	-0.074 (-0.185, 0.038)	0.102 (-0.018, 0.221)	0.072 (-0.1033, 0.246)	0.0459
Change in CMRS						
Participants	1179	1331	1066	798	300	

β (95% CI), Model 1	0.112 (-0.061, 0.284)	0.195 (0.014, 0.376)	0.040 (-0.156, 0.236)	-0.182 (-0.461, 0.098)	0.0578
β (95% CI), Model 2	0.104 (-0.062, 0.270)	0.134 (-0.039, 0.306)	0.044 (-0.141, 0.229)	-0.211 (-0.476, 0.054)	0.1009

BMI, body mass index; CI, confidence interval; CMRS, cardiometabolic risk score; DBP, diastolic blood pressure; HOMA-IR, homeostatic model assessment of insulin resistance; HDL-C, high-density lipoprotein cholesterol; LDL-C, low-density lipoprotein cholesterol; MAP, mean arterial pressure; SBP, systolic blood pressure; TC, total cholesterol; TG, triglyceride.

*GLM was used to estimate multivariable-adjusted β (95% CI) of cardiometabolic risk score between quintiles of energy intake from different meals with the quintile 1 as the reference.

†Model 1 was adjusted for classes in school as clustering effects and characteristics of individuals including age, sex, and corresponding CMR factor at baseline as fixed effects.

‡Model 2 was adjusted for Model 1 plus puberty, grade, intervention, puberty, BMI, physical activity, and total energy intake.

Table S6. Changes in macronutrients intake at different meals and change in cardiometabolic risk score

	Consumption level					
	Quintile 1	Quintile 2	Quintile 3	Quintile 4	Quintile 5	P-trend*
Change in carbohydrate at breakfast						
Range (%)	<-16.24	-16.24, -4.56	-4.57, 4.46	4.47, 16.53	>16.53	
Participants	700	687	712	693	708	
β (95% CI), Model 1†		0.001 (-0.228, 0.230)	-0.071 (-0.303, 0.161)	-0.051 (-0.291, 0.188)	-0.289 (-0.565, -0.013)	0.07
β (95% CI), Model 2‡		-0.049 (-0.268, 0.170)	-0.037 (-0.259, 0.185)	-0.055 (-0.284, 0.175)	-0.238 (-0.502, 0.026)	0.15
β (95% CI), Model 3§		-0.059 (-0.278, 0.160)	-0.044 (-0.266, 0.180)	-0.056 (-0.285, 0.174)	-0.235 (-0.499, 0.029)	0.16
Change in carbohydrate at lunch						
Range (%)	<-23.48	-23.48, -9.87	-9.87, 1.96	1.97, 15.07	>15.07	
Participants	717	710	687	689	697	
β (95% CI), Model 1		0.023 (-0.204, 0.250)	0.208 (-0.032, 0.448)	0.304 (0.055, 0.554)	0.352 (0.063, 0.641)	0.0029
β (95% CI), Model 2		0.024 (-0.193, 0.241)	0.182 (-0.048, 0.411)	0.277 (0.037, 0.516)	0.325 (0.048, 0.602)	0.0045
β (95% CI), Model 3		0.015 (-0.202, 0.232)	0.186 (-0.044, 0.415)	0.269 (0.030, 0.508)	0.308 (0.032, 0.585)	0.0061
Change in carbohydrate at dinner						
Range (%)	<-23.89	-23.89, -8.93	-8.94, 3.23	3.24, 17.85	>17.85	
Participants	716	686	711	693	694	

β (95% CI), Model 1		-0.072 (-0.303, 0.158)	-0.029 (-0.261, 0.204)	0.016 (-0.231, 0.264)	0.055 (-0.230, 0.339)	0.57
β (95% CI), Model 2		-0.081 (-0.301, 0.139)	-0.018 (-0.240, 0.205)	-0.018 (-0.255, 0.219)	0.141 (-0.132, 0.414)	0.33
β (95% CI), Model 3		-0.086 (-0.306, 0.134)	-0.024 (-0.246, 0.199)	-0.024 (-0.261, 0.213)	0.131 (-0.143, 0.404)	0.38
Change in protein at lunch						
Range (%)	<-5.62	-5.62, -0.75	-0.76, 2.89	2.90, 7.86	>7.86	
Participants	668	696	722	699	715	
β (95% CI), Model 1		-0.262 (-0.506, -0.019)	-0.402 (-0.660, -0.145)	-0.554 (-0.818, -0.290)	-0.642 (-0.922, -0.362)	<0.0001
β (95% CI), Model 2		-0.245 (-0.477, -0.012)	-0.4044 (-0.6508, -0.1580)	-0.5531 (-0.8059, -0.3003)	-0.6959 (-0.9636, -0.4281)	<0.0001
β (95% CI), Model 3		-0.2330 (-0.4654, -0.0005)	-0.393 (-0.641, -0.146)	-0.532 (-0.786, -0.279)	-0.685 (-0.953, -0.416)	<0.0001
Change in protein at dinner						
Range (%)	<-7.18	-7.18, -1.44	-1.45, 3.12	3.13, 8.75	>8.75	
Participants	703	705	698	689	705	
β (95% CI), Model 1		0.028 (-0.218, 0.273)	-0.127 (-0.385, 0.131)	-0.333 (-0.598, -0.068)	-0.155 (-0.431, 0.121)	0.0277
β (95% CI), Model 2		0.014 (-0.221, 0.249)	-0.180 (-0.427, 0.067)	-0.309 (-0.563, -0.055)	-0.1882(-0.453, 0.077)	0.0188
β (95% CI), Model 3		0.007 (-0.228, 0.242)	-0.178 (-0.426, 0.070)	-0.309 (-0.563, -0.055)	-0.185 (-0.450, 0.081)	0.0222
Change in fat at breakfast						
Range (%)	<-13.78	-13.78, -4.17	-4.18, 2.95	2.95, 12.45	>12.45	

Participants	704	698	711	687	700	
β (95% CI), Model 1		0.100 (-0.139, 0.339)	0.136 (-0.115, 0.388)	0.245 (-0.014, 0.504)	0.262 (-0.015, 0.538)	0.0362
β (95% CI), Model 2		0.102 (-0.127, 0.330)	0.094 (-0.147, 0.334)	0.200 (-0.048, 0.448)	0.206 (-0.058, 0.471)	0.0966
β (95% CI), Model 3		0.095 (-0.133, 0.324)	0.0845 (-0.1556, 0.3247)	0.1898 (-0.0580, 0.4376)	0.2110 (-0.0532, 0.4753)	0.0904
Change in fat at lunch						
Range (%)	<-12.71	-12.71, -2.24	-2.24, 7.50	7.51, 18.75	>18.75	
Participants	687	696	700	697	720	
β (95% CI), Model 1		0.023 (-0.224, 0.270)	0.030(-0.235, 0.295)	0.061 (-0.215, 0.338)	-0.083 (-0.377, 0.212)	0.62
β (95% CI), Model 2		-0.017 (-0.254, 0.219)	0.045 (-0.208, 0.299)	-0.009 (-0.274, 0.256)	-0.038 (-0.319, 0.245)	0.79
β (95% CI), Model 3		-0.010 (-0.246, 0.227)	0.049 (-0.204, 0.303)	-0.0002 (-0.265, 0.264)	-0.029 (-0.312, 0.253)	0.83

*GLM was used to estimate multivariable-adjusted means and standard errors of cardiometabolic risk score between quintiles of macronutrient intake from different meals with the quintile 1 as the reference.

†Model 1 was adjusted for classes in school as clustering effects and characteristics of individuals including age, sex, and CMRS at baseline as fixed effects.

‡Model 2 was adjusted for Model 1 plus puberty, grade, intervention, puberty, BMI, physical activity, and total energy intake.

§Model 3 was adjusted for Model 2 plus birthweight, household income, mother's education, father's education, mother's BMI, and father's BMI as fixed effects.

Table S7. Meal patterns and changes in cardiometabolic risk factors in children in the control group

	Meal pattern					P-value
	Balanced	Breakfast dominant	Lunch dominant	Dinner dominant	Snack dominant	
Change in BMI						
Participants	640	675	591	421	162	
β (95% CI), Model 1[†]		0.014 (-0.051, 0.079)	0.036 (-0.030, 0.105)	0.016 (-0.058, 0.090)	-0.060 (-0.165, 0.044)	0.47
β (95% CI), Model 2[‡]		0.034 (-0.032, 0.100)	0.050 (-0.017, 0.117)	0.020 (-0.053, 0.093)	-0.063 (-0.166, 0.041)	0.23
β (95% CI), Model 3[§]		0.033 (-0.032, 0.099)	0.047 (-0.020, 0.114)	0.021 (-0.052, 0.094)	-0.058 (-0.161, 0.046)	0.30
Change in WC						
Participants	638	676	592	421	163	
β (95% CI), Model 1		-0.013 (-0.065, 0.038)	0.025 (-0.028, 0.078)	0.010 (-0.048, 0.068)	-0.022 (-0.104, 0.060)	0.63
β (95% CI), Model 2		0.008 (-0.043, 0.058)	0.036 (-0.016, 0.088)	0.016 (-0.041, 0.072)	-0.036 (-0.115, 0.044)	0.43
β (95% CI), Model 3		0.007 (-0.044, 0.058)	0.032 (-0.020, 0.083)	0.019 (-0.037, 0.076)	-0.027 (-0.107, 0.052)	0.58
Change in PBF						
Participants	623	656	574	411	159	
β (95% CI), Model 1		0.054 (-0.020, 0.128)	-0.003 (-0.079, 0.073)	-0.076 (-0.159, 0.007)	-0.027 (-0.145, 0.091)	0.0425
β (95% CI), Model 2		0.038 (-0.035, 0.112)	-0.004 (-0.079, 0.071)	-0.090 (-0.172, -0.009)	-0.056 (-0.172, 0.060)	0.0327
β (95% CI), Model 3		0.042 (-0.032, 0.116)	-0.0001 (-0.075, 0.075)	-0.089 (-0.171, -0.007)	-0.052 (-0.169, 0.064)	0.0301
Change in SBP						

Participants	637	679	591	420	162	
β (95% CI), Model 1		0.127 (0.020, 0.235)	0.076 (-0.036, 0.187)	0.062 (-0.059, 0.183)	-0.006 (-0.178, 0.167)	0.18
β (95% CI), Model 2		0.108 (0.002, 0.213)	0.057 (-0.051, 0.165)	0.050 (-0.067, 0.168)	-0.021 (-0.188, 0.146)	0.30
β (95% CI), Model 3		0.109 (0.003, 0.214)	0.062 (-0.045, 0.170)	0.060 (-0.057, 0.178)	-0.023 (-0.190, 0.144)	0.27
Change in DBP						
Participants	638	678	592	420	162	
β (95% CI), Model 1		0.121 (0.015, 0.228)	-0.030 (-0.140, 0.080)	0.0005 (-0.120, 0.121)	0.044 (-0.127, 0.215)	0.06
β (95% CI), Model 2		0.088 (-0.018, 0.194)	-0.049 (-0.157, 0.060)	-0.011 (-0.129, 0.107)	0.033 (-0.135, 0.202)	0.15
β (95% CI), Model 3		0.077 (-0.029, 0.182)	-0.051 (-0.159, 0.057)	-0.004 (-0.122, 0.114)	0.027 (-0.140, 0.195)	0.22
Change in MAP						
Participants	637	678	591	420	162	
β (95% CI), Model 1		0.139 (0.031, 0.247)	0.012 (-0.100, 0.124)	0.027 (-0.095, 0.148)	0.031 (-0.142, 0.204)	0.09
β (95% CI), Model 2		0.108 (0.001, 0.215)	-0.008 (-0.117, 0.101)	0.015 (-0.104, 0.133)	0.018 (-0.151, 0.187)	0.22
β (95% CI), Model 3		0.100 (-0.006, 0.207)	-0.007 (-0.116, 0.102)	0.024 (-0.095, 0.142)	0.013 (-0.156, 0.181)	0.29
Change in TC						
Participants	604	631	549	399	149	
β (95% CI), Model 1		-0.065 (-0.138, 0.008)	0.014 (-0.062, 0.090)	-0.115 (-0.198, -0.033)	0.0782(-0.041, 0.197)	0.0024
β (95% CI), Model 2		-0.049 (-0.123, 0.025)	0.022 (-0.054, 0.098)	-0.114 (-0.196, -0.032)	0.075 (-0.044, 0.194)	0.0037
β (95% CI), Model 3		-0.049 (-0.123, 0.025)	0.026 (-0.049, 0.102)	-0.117 (-0.199, -0.035)	0.076 (-0.044, 0.195)	0.0024

Change in HDL-C						
Participants	604	634	549	399	148	
β (95% CI), Model 1		0.054 (-0.051, 0.158)	-0.006 (-0.114, 0.103)	-0.082 (-0.120, 0.036)	0.261 (0.090, 0.432)	0.0041
β (95% CI), Model 2		0.063 (-0.042, 0.167)	-0.004 (-0.111, 0.103)	-0.072 (-0.187, 0.044)	0.283 (0.115, 0.451)	0.0017
β (95% CI), Model 3		0.060 (-0.045, 0.165)	0.003 (-0.104, 0.110)	-0.065 (-0.181, 0.051)	0.270 (0.102, 0.438)	0.0044
Change in LDL-C						
Participants	604	632	549	399	150	
β (95% CI), Model 1		-0.130 (-0.211, -0.050)	-0.049 (-0.133, 0.034)	-0.106 (-0.196, -0.015)	-0.062 (-0.192, 0.068)	0.02
β (95% CI), Model 2		-0.097 (-0.178, -0.016)	-0.039 (-0.122, 0.044)	-0.104 (-0.194, -0.014)	-0.068 (-0.198, 0.061)	0.09
β (95% CI), Model 3		-0.094 (-0.175, -0.014)	-0.032 (-0.114, 0.051)	-0.104 (-0.194, -0.015)	-0.054 (-0.183, 0.075)	0.09
Change in TG						
Participants	603	633	550	400	150	
β (95% CI), Model 1		-0.042 (-0.141, 0.057)	-0.0317 (-0.133, 0.072)	-0.0250(-0.136, 0.086)	-0.147 (-0.308, 0.013)	0.50
β (95% CI), Model 2		-0.053 (-0.152, 0.045)	-0.032 (-0.133, 0.069)	-0.034 (-0.143, 0.075)	-0.172 (-0.329, -0.014)	0.31
β (95% CI), Model 3		-0.056 (-0.155, 0.043)	-0.036 (-0.137, 0.065)	-0.027 (-0.136, 0.082)	-0.162 (-0.319, -0.004)	0.35
Change in fasting glucose						
Participants	604	633	549	399	150	
β (95% CI), Model 1		-0.074 (-0.156, 0.008)	-0.106 (-0.191, -0.022)	0.009 (-0.082, 0.101)	-0.049 (-0.182, 0.083)	0.0504
β (95% CI), Model 2		-0.071 (-0.154, 0.012)	-0.108 (-0.193, -0.023)	0.012 (-0.080, 0.103)	-0.041 (-0.174, 0.091)	0.0488

β (95% CI), Model 3		-0.073 (-0.156, 0.011)	-0.110 (-0.195, -0.025)	0.010 (-0.082, 0.102)	-0.037 (-0.171, 0.096)	0.0478
Change in insulin						
Participants	548	559	482	360	137	
β (95% CI), Model 1		0.064 (-0.104, 0.231)	-0.068 (-0.245, 0.106)	0.200 (0.012, 0.388)	0.048 (-0.221, 0.317)	0.0885
β (95% CI), Model 2		-0.038 (-0.202, 0.127)	-0.124 (-0.293, 0.046)	0.198 (0.016, 0.380)	-0.003 (-0.263, 0.256)	0.0181
β (95% CI), Model 3		-0.029 (-0.195, 0.136)	-0.134 (-0.303, 0.035)	0.193 (0.011, 0.375)	-0.001 (-0.262, 0.260)	0.0178
Change in HOMA-IR						
Participants	548	559	481	360	137	
β (95% CI), Model 1		0.060 (-0.103, 0.223)	-0.094 (-0.262, 0.075)	0.187 (0.005, 0.368)	0.0549 (-0.2051, 0.315)	0.0538
β (95% CI), Model 2		-0.039 (-0.199, 0.120)	-0.148 (-0.312, 0.016)	0.184 (0.008, 0.360)	0.006 (-0.246, 0.258)	0.0099
β (95% CI), Model 3		-0.033 (-0.193, 0.127)	-0.160 (-0.324, 0.004)	0.178 (0.001, 0.354)	0.008 (-0.245, 0.261)	0.0091
Change in CMRS						
Participants	559	569	500	358	140	
β (95% CI), Model 1		-0.070 (-0.323, 0.183)	-0.073 (-0.334, 0.188)	0.088 (-0.196, 0.372)	-0.357 (-0.7621, 0.047)	0.33
β (95% CI), Model 2		-0.086 (-0.331, 0.158)	-0.077 (-0.326, 0.172)	0.102 (-0.168, 0.373)	-0.482 (-0.869, -0.096)	0.08
β (95% CI), Model 3		-0.107 (-0.352, 0.137)	-0.102 (-0.352, 0.147)	0.095 (-0.176, 0.367)	-0.482 (-0.868, -0.095)	0.07

BMI, body mass index; CI, confidence interval; CMRS, cardiometabolic risk score; DBP, diastolic blood pressure; HOMA-IR, homeostatic model assessment of insulin resistance; HDL-C, high-density lipoprotein cholesterol; LDL-C, low-density lipoprotein cholesterol; MAP, mean arterial pressure; SBP, systolic blood pressure; TC, total cholesterol; TG, triglyceride.

*GLM was used to estimate multivariable-adjusted means and standard errors of cardiometabolic risk factors between-meal patterns with the balanced meal patterm as the referemce. We used the Benjamin-Hochberg procedure to control the false discovery rate at level 5% for multiple comparisons with the P-value cut-off point of significance was 0.0107.

†Model 1 was adjusted for classes in school as clustering effects and characteristics of individuals including age, sex, and corresponding CMR factor at baseline as fixed effects.

‡Model 2 was adjusted for Model 1 plus puberty, grade, intervention, BMI, physical activity, and intake of energy, fiber, vegetable, fruit, pork, legumes, and nuts at baseline as fixed effects.

§Model 3 was adjusted for Model 2 plus birthweight, household income, mother's education, father's education, mother's BMI, and father's BMI as fixed effects.
